# Cardiovascular autonomic function testing in multiple system atrophy and Parkinson’s disease: an expert-based blinded evaluation

**DOI:** 10.1007/s10286-020-00691-4

**Published:** 2020-05-15

**Authors:** Fabian Leys, Alessandra Fanciulli, Jean-Pierre Ndayisaba, Roberta Granata, Walter Struhal, Gregor K. Wenning

**Affiliations:** 1 Division of Clinical Neurobiology, Department of Neurology, Medical University of Innsbruck, Innsbruck, Austria; 2 Department of Neurology, University Clinic Tulln, Karl Landsteiner University of Health Sciences, Tulln, Austria

**Keywords:** Parkinson’s disease, Multiple system atrophy, Orthostatic hypotension, Cardiovascular autonomic failure, Cardiovascular autonomic function testing

## Abstract

**Purpose:**

Multiple system atrophy (MSA) and Parkinson’s disease (PD) are sporadic neurodegenerative diseases characterized by an accumulation of misfolded α-synuclein. Cardiovascular autonomic failure develops in both MSA and PD, although studies indicate different sites of autonomic nervous system lesion. However, it is unclear whether this could potentially aid the differential diagnosis of these diseases. Here we determined whether cardiovascular autonomic function testing (CAFT) can discriminate between the parkinsonian variant of MSA (MSA-P) and PD based on either an expert-based blinded evaluation or a systematic comparison of cardiovascular autonomic function indices.

**Methods:**

We included 22 patients aged 55–80 with neurogenic orthostatic hypotension (nOH) who had been diagnosed with either clinically probable MSA-P (*n* = 11) according to current consensus criteria or clinically definite PD (*n* = 11) according to the Queen Square criteria. Three physicians with expertise in CAFT were blinded to the neurological diagnosis and were asked to identify the correct neurological diagnosis by applying a self-created evaluation scheme to the CAFT recordings. Afterwards, a systematic comparison of clinical–demographic characteristics and CAFT parameters was carried out.

**Results:**

Neither the raters (overall diagnostic accuracy: 58.46%) nor the evaluation scheme created post hoc (72.73%) showed reliable discriminatory capacity. The inter-rater reliability was slight (*κ* = 0.01). We observed no statistically significant differences in cardiovascular autonomic indices between PD and MSA-P patients.

**Conclusion:**

CAFT is the gold standard for assessing the presence and severity of cardiovascular autonomic failure, but the results of our pilot study suggest that CAFT might be of limited value in the differential diagnosis between MSA-P and PD once nOH is present.

## Introduction

Multiple system atrophy (MSA) and Parkinson’s disease (PD) are sporadic neurodegenerative disorders characterized by an accumulation of misfolded α-synuclein [1, 2]. It is a major clinical challenge to distinguish between MSA and PD at disease onset due to their multifaceted presentation and overlapping features, including cardiovascular autonomic failure [1, 3–5]. Orthostatic hypotension (OH) is a cardinal sign of autonomic failure and is, by consensus, defined as a sustained reduction in systolic blood pressure (BP) of at least 20 mmHg or diastolic BP of at least 10 mmHg within 3 min of standing or head-up tilt [6, 7]. When OH is caused by primary autonomic degenerative disorders (e.g. MSA, PD) or is secondary to systemic diseases such as diabetes or amyloidosis, the condition is called neurogenic orthostatic hypotension (nOH) [8], which is best differentiated from other causes of BP instability by checking for missing BP counterregulation during the Valsalva maneuver or an absent or insufficient heart rate (HR) increase upon tilt or standing despite severe BP falls [9, 10]. A meta-analysis by Velseboer et al. [11] reported an OH prevalence of 30% in PD patients, with high heterogeneity between the studies analyzed, whereas OH is present in 57–78% of MSA cases [4, 12]. Neuropathological studies [13, 14] have shown that the site of the autonomic nervous system lesion is predominantly central in MSA and peripheral in PD. However, as different conclusions have been drawn from different studies, possibly due to different prevalences of overt cardiovascular autonomic failure, it is still unclear whether MSA and PD can be distinguished by means of cardiovascular autonomic function testing (CAFT) once OH is present [10, 15–32]. Therefore, we aimed to determine whether CAFT can discriminate between nOH due to the parkinsonian variant of MSA (MSA-P) and nOH due to PD based on either an expert-based blinded evaluation or a systematic comparison of cardiovascular autonomic parameters. In contrast to an objective statistical analysis, the expert-based blinded evaluation was performed to provide novel insight into whether the interplay of rater experience and CAFT findings can distinguish between MSA-P and PD in a clinical–practical context.

## Methods

### Study population and data set

We retrospectively included 22 parkinsonian patients who had undergone CAFT between October 2007 and May 2018. Among these patients, 11 had probable MSA-P diagnosed according the second consensus criteria [33] and 11 had clinically definite PD diagnosed according to the Queen Square criteria [5]. Inclusion criteria were: (1) age between 55 and 80 years; (2) CAFT including the Valsalva maneuver and deep breathing; (3) sufficient record quality; (4) either probable MSA-P [33] or clinically definite PD [5]; (5) presence of OH as defined by consensus [6]; (6) missing BP overshoot during phase IV of the Valsalva manoeuvre [9] or nOH as defined by Norcliffe-Kaufmann et al. [10].

The following features were considered exclusion criteria: (1) secondary cause of parkinsonism as detected by history or investigation; (2) OH of non-neurogenic origin; (3) other major neurologic or psychiatric disorders that significantly interfere with the clinical presentation (e.g. severe polyneuropathy, dementia, major depressive or psychotic disorders according to DSM-V); (4) diabetes mellitus; (5) incomplete clinical information available.

### Cardiovascular autonomic function testing

The standard protocol in our cardiovascular autonomic function unit consisted of 10 min supine, 10 min of 60° head-up tilt, 5 min supine, and 5 min of active standing [34]. In addition, the patients performed the Valsalva maneuver and deep breathing. According to our standard operating procedures, patients underwent examinations in a quiet room with a constant temperature of approximately 22 °C, provided they were on regular medication and had received instructions not to drink any coffee, tea, or taurine-containing beverages on the day of the examination and to fast for at least 2 h before testing. HR and BP were continuously recorded via noninvasive beat-to-beat finger-cuff BP recording and impedance cardiography (Task Force^®^ Monitor, TFM) as well as by oscillometric arm cuff BP measurements. In the present study, HR and BP values at the 10th minute supine, the 3rd and 10th minutes upon tilting, the 5th minute supine, and the 3rd and 5th minutes upon standing were calculated by averaging 15 values of the continuous HR and BP recordings at the above given time points. The expiratory/inspiratory ratio (*E*/*I* ratio) was calculated as the mean of six ratios that were obtained by dividing the longest *R*–*R* interval during expiration by the shortest *R*–*R* interval during inspiration in the electrocardiography recording. The Valsalva ratio was calculated by dividing the highest HR in phase II of the Valsalva maneuver by the lowest HR in phase IV. BP counterregulatory behavior during the Valsalva maneuver was calculated according to a standardized methodology described elsewhere [35].

### Blinded assessment

Three raters with expertise in CAFT (AF, RG, WS) were blinded to the neurological diagnosis of the patient vignettes and asked to assign the correct diagnosis (i.e., MSA or PD) by applying a self-created evaluation scheme to the CAFT records. The blinded assessment was structured as follows: (A) work instructions; (B) age-related reference values for the Valsalva maneuver and deep breathing [36]; (C) 22 patient vignettes, including the patient’s (1) gender and (2) age at the time of CAFT; oscillometric HR and BP values during (3) the head-up tilt test and (4) the standing test; (5) the Valsalva ratio and systolic/diastolic BP values at phase I, early and late phase II, phase III, and phase IV of the Valsalva maneuver; (6) the *E*/*I* ratio; as well as (7) a printout of the continuous HR and BP trends monitored by the TFM during the examination. At the end, the raters were given a free-text form that they used to summarize the evaluation schemes they created.

Based on personal experience, the literature, and the hypothesis that autonomic failure is more severe in MSA than in PD, rater #1 created an evaluation scheme that included a six-item probability score system to identify MSA-P along with an additional factor suggesting PD (Table 1A). The design of rater #2′s evaluation scheme to identify MSA was based on the literature [10, 19, 20, 24, 26, 32, 37] and personal experience in CAFT (Table 1B). The main rationale behind rater #3′s evaluation scheme to distinguish between MSA and PD was that baroreflex sensitivity is more blunted in PD than in MSA (Table 1C) [31]. Moreover, if the 3 min 20/10 mmHg BP fall criterion for OH [6] was not met based on oscillometric measurements, but under continuous BP monitoring, rater #3 used this to definitely exclude the diagnosis of MSA.

**Table 1:** Blinded assessment: raters’ evaluation schemes

A	B	C
Rater #1: six-item PSS for diagnosis of MSA-P	Rater #2: five-item PSS for diagnosis of MSA-P	Rater #3: three-item PSS to distinguish between MSA-P and PD
Age < 65 Supine HR > 70 bpm Moderate/severe nSH [40] Orthostatic BP fall > 30/15 mmHg Missing BP overshoot at late phase II and phase IV of Valsalva maneuver Pathological VR and DB Additional factor suggesting PD: supine BP < 110/70 mmHg	Supine HR > 75 bpm Supine BP > 140/90 mmHg Pathological VR Abnormal HR variation during head-up tilt and Valsalva maneuver Orthostatic BP fall does not exceed > 40/20 mmHg	Baroreflex sensitivity: phase IV of Valsalva maneuver in PD more blunted than in MSA-P No OH in oscillometric BP measurements = no MSA-P Slow progressive changes hint at pharmacological influence

A) Diagnosis of MSA-P was assigned if  score > 3 points. −1 point was assigned if the additional factor suggesting PD was positive

B) Diagnosis was made by considering the applicable items of the evaluation scheme together with the rater’s subjective impression of the patient’s vignette*

C) Diagnosis was made by considering the applicable items of the evaluation scheme together with the rater’s subjective impression of the patient’s vignette*

*PSS* probability score system, *MSA-P* parkinsonian variant of multiple system atrophy, *PD* Parkinson’s disease, *HR* heart rate, *nSH* neurogenic supine hypertension, *BP* blood pressure, *VR* Valsalva ratio, *DB* deep breathing, *OH* orthostatic hypotension

*Raters #2 and #3 did not apply a strict cutoff value

### Statistical analysis

Qualitative variables were summarized by the relative and absolute frequencies, and quantitative variables by the median and the 25–75th percentiles. We used the Shapiro–Wilk test to test for normality. Qualitative variables were compared by means of chi-squared or Fisher’s exact test, whereas quantitative variables were compared using the Mann–Whitney *U* test if the variables were non-normally distributed or the *T* test if they were Gaussian distributed. We applied a post-hoc Bonferroni correction to multiple tests [38]. A two-tailed *p* < 0.05 was considered statistically significant. Statistical analysis was performed by means of IBM SPSS^®^ Statistics v.24.0 (IBM Corporation, Armonk, NY, USA). Diagnostic accuracy was calculated in Microsoft^®^ Office Excel 2016 (Microsoft Corporation, Redmond, WA, USA) as the sum of the true positive and true negative rates divided by the total sample size. Inter-rater reliability was calculated according to Fleiss’* κ* for multiple raters [39].

## Results

### Clinical demographic parameters

An overview of the patients’ clinical–demographic characteristics is shown in Table 2. Patients with PD had a median age of 70 (65; 75) years that was marginally, but not significantly (*p* = 0.102), older than patients with MSA-P [67 (60; 72) years]. There was an excess in the male-to-female ratio in both groups (10:1 in PD versus 8:3 in MSA-P; *p* = 0.586). Disease severity, measured on the Hoehn and Yahr scale, was significantly higher in patients with MSA-P [4 (2.5; 4)] compared to patients with PD [2 (2; 2.5); *p* = 0.008], while the disease durations in the groups were comparable [PD 4 (4; 8) versus MSA-P 3 (2; 5); *p* = 1.000]. All patients except for one in the MSA-P group (*n* = 21; 95.50%) had orthostatic complaints at the time of CAFT (*p* = 1.000). Cardiovascular comorbidities were present in seven PD (*n* = 7; 63.60%) and four MSA-P (*n* = 4; 36.40%) patients, which did not correspond to a significant difference (*p* = 0.395). The cohorts did not differ statistically with respect to dopaminergic or other therapies.

**Table 2:** Clinical–demographic parameters

Features	PD	MSA-P	*p* value
Number of patients	11	11	
Age, years	70 (65; 75)	67 (60; 72)	0.102
Age < 65 years	18% (2)	46% (5)	0.361
Gender, male	91% (10)	73% (8)	0.586
Age at disease onset, years	64 (58; 70)	62 (56; 68)	0.513
Hoehn and Yahr stage	2 (2; 2.5)	4 (2.5; 4)	0.008
Disease duration, years	4 (4; 8)	3 (2; 5)	1.000
Follow-up time, months	26 (16; 90)	10 (1; 22)	0.395
Orthostatic symptoms at CAFT time	100% (11)	91% (10)	1.000
Cardiovascular comorbidities	64% (7)	36% (4)	0.395
Parkinsonian phenotype			0.087
Tremor dominant	9% (1)	0% (0)	
Akinetic rigid	64% (7)	100% (11)	
Mixed type	27% (3)	0% (0)	
Drug intake			
Total number of drugs	6 (3; 9)	7 (5; 8)	0.784
Use of levodopa	64% (7)	91% (10)	0.311
Daily levodopa dosage, mg/day	300 (0; 600)	600 (400; 800)	0.087
Use of dopamine agonists	36% (4)	18% (2)	0.635
DA-LED, mg/day	0 (0; 120)	0 (0; 0)	0.635
LEDD, mg/day	300 (120; 873)	759 (400; 1,075)	0.170
Use of antihypotensive drugs	18% (2)	36% (4)	0.635
Use of antihypertensive drugs	27% (3)	46% (5)	0.659

Categorical variables are characterized as absolute and relative (%, in parentheses) values; continuous variables are characterized as the median value and the 25–75th percentiles (in pathentheses)

*PD* Parkinson’s disease, *MSA-P* parkinsonian variant of multiple system atrophy, *CAFT* cardiovascular autonomic function testing, *DA-LED* levodopa equivalent dose of dopamine agonist, *LEDD* levodopa equivalent daily dose

### Blinded rater assessment

The results of the blinded assessment are provided in Table 3. The inter-rater reliability, calculated as Fleiss’* κ* [39], was 0.01, indicating slight agreement. During the blinded assessment, nine of the 22 cases received the same diagnosis by the raters. Among those nine cases, the raters correctly assigned a diagnosis of PD to six cases and misdiagnosed three MSA-P cases. None of the MSA-P patients were correctly identified by all the raters. No discriminatory pattern of cardiovascular parameters between MSA and PD patients was found in a manual analysis of those nine cases. We further calculated the sensitivity, specificity, positive predictive value (PPV), negative predictive value (NPV), and diagnostic accuracy of each item in each rater’s evaluation scheme and investigated whether combining the most accurate items in each scheme would increase the diagnostic yield, but this did not improve the diagnostic power in any of the rater’s schemes (Table 4). However, an evaluation scheme that was created post hoc and combined the four most accurate diagnostic items overall (supine HR > 70 bpm, supine BP > 140/90 mmHg, age < 65 years, abnormal HR variation during head-up tilt and Valsalva maneuver) with an additional factor suggesting PD (supine BP < 110/70 mmHg, corresponding to − 1 point) allowed MSA-P to be identified with 45.46% sensitivity, 100.00% specificity, 100.00% PPV, 64.71% NPV, and 72.73% diagnostic accuracy when a score of ≥ 2 was assumed to indicate MSA-P.

**Table 3:** Diagnostic accuracy of the blinded assessment and of the evaluation scheme created post hoc

	Sensitivity	Specificity	PPV	NPV	Diagnostic accuracy
Rater #1	45.46	72.73	62.50	57.14	59.09
Rater #2	27.27	100.00	100.00	57.89	63.64
Rater #3^*^	30.00	72.73	50.00	53.33	52.38
Overall	34.38	81.82	64.71	56.25	58.46
Evaluation scheme created post hoc	45.46	100.00	100.00	64.71	72.73

Values are number (%)

*PPV* positive predictive value, *NPV* negative predictive value

*21 out of 22 cases (10 MSA-P, 11 PD) were evaluated; case #8 was not evaluated due to artefact overlays in the Task Force^®^ Monitor records

**Table 4:** Analysis of the ability of each item in the raters’ schemes to identify MSA-P (test positive) and exclude PD (test negative)

	Sensitivity	Specificity	PPV	NPV	Diagnostic accuracy
Rater #1					
Age < 65 years	45.46	81.82	71.43	60.00	63.64
Supine HR > 70 bpm*	36.36	100.00	100.00	61.11	68.18
Moderate/severe nSH [40]*	27.27	90.91	75.00	55.56	59.09
Orthostatic BP fall > 30/15 mmHg*	27.27	72.73	50.00	50.00	50.00
Missing BP overshoot at late phase II and phase IV of Valsalva maneuver	90.91	27.27	55.56	75.00	59.09
Pathological VR and DB	45.46	63.64	55.56	53.84	54.55
Supine BP < 110/70 mmHg suggests PD*	90.91	18.18	52.63	66.67	54.55
Combination of “age < 65 years” and “supine HR > 70 bpm*”	27.27	100.00	100.00	57.89	63.64
Rater #2					
Supine HR > 75 bpm*	27.27	100.00	100.00	57.89	63.64
Supine BP > 140/90 mmHg*	45.46	90.91	83.33	62.50	68.18
Pathological VR	54.55	63.64	60.00	58.33	59.09
Abnormal HR variation during head-up tilt and Valsalva maneuver	27.27	100.00	100.00	57.89	63.64
Orthostatic BP fall does not exceed > 40/20 mmHg*	81.82	18.18	50.00	50.00	50.00
Combination of “supine HR > 75 bpm*” and “supine BP > 140/90 mmHg*”	27.27	100.00	100.00	57.89	63.64
Combination of “supine HR > 75 bpm*” and “abnormal HR variation during head-up tilt and Valsalva maneuver”	9.09	100.00	100.00	52.38	54.55
Combination of “abnormal HR variation during head-up tilt and Valsalva maneuver” and “supine BP > 140/90 mmHg*”	18.18	100.00	100.00	55.00	59.09
Rater #3					
Baroreflex sensitivity: phase IV of Valsalva maneuver in PD more blunted than in MSA-P	30.00	72.73	50.00	53.33	52.38
No OH in oscillometric BP measurements equals no MSA-P	100.00	9.09	50.00	100.00	52.38
Slow progressive changes hint at pharmacological influence	30.00	81.82	60.00	56.25	57.14

Values are number (%)

*MSA-P* parkinsonian variant of multiple system atrophy, *PD* parkinson's disease, *PPV* positive predictive value, *NPV* negative predictive value, *HR* heart rate, *nSH* neurogenic supine hypertension, *BP* blood pressure, *VR* valsalva ratio, *DB* deep breathing, *OH* orthostatic hypotension

*Calculated based on cardiovascular parameters of the head-up tilt

### Cardiovascular autonomic function indices

Six of the 22 patients (*n* = 6; 27.30%) had mild to severe neurogenic supine hypertension (nSH) [40] during CAFT, measured at the end of the 10 min supine phase before head-up tilt. Only one patient with nSH (*n* = 1; 16.70%) belonged to the PD group, but this did not correspond to a significant difference between the groups (*p* = 0.149). A systematic comparison of cardiovascular parameters of the head-up tilt, active standing test, Valsalva maneuver, and deep breathing between the groups did not discern a significant difference in any of the analyzed variables (Table 5). Systolic and diastolic BP falls after 3 min of active standing seemed to be slightly more severe in MSA-P than in PD, whereas they were almost equal upon head-up tilt. Analysis of the parameter “supine HR” revealed a trend towards a statistically significant difference between groups, with median heart rates of 62 (54; 67) bpm in the PD group and 68 (58; 76) bpm in the MSA-P group at the 10th minute supine before head-up tilt (*p* = 0.037) and 59 (54; 65) bpm in the PD group and 67 (64; 73) bpm in the MSA-P group at the 5th minute supine before the standing test (*p* = 0.033), but these results did not withstand the post hoc Bonferroni correction [38]. Analysis of the parameter “supine HR > 70,” which was also part of rater #1′s 6-item probability score system, showed that none of the PD patients (*n* = 0; 0.00%) but four of the MSA-P patients (*n* = 4; 36.40%) featured a heart rate of > 70 bpm before head-up tilt. Nevertheless, this did not correspond to a significant difference between groups (*p* = 0.090). Indices of parasympathetic cardiac control (as reflected in the *E*/*I* ratio and the Valsalva ratio) as well as indices of sympathetic function (as reflected in the BP and HR changes evoked by head-up tilt and the BP changes during the late phase II and phase IV of the Valsalva maneuver) did not show any significant difference between the PD and MSA patients (*p* > 0.05).

**Table 5:** Statistical analysis of the cardiovascular autonomic function indices

Cardiovascular parameters	PD	MSA-P	*p* value
nSH [40]	9.10% (1)	45.50% (5)	0.149
Mild nSH [> 140/90 mmHg]	0% (0)	40.00% (2)	
Moderate nSH [> 160/100 mmHg]	0% (0)	40.00% (2)	
Severe nSH [> 180/110 mmHg]	100.00% (1)	20.00% (1)	
Head-up tilt			
Supine HR, bpm	62 (54; 67)	68 (58; 76)	0.037
Supine HR > 70 bpm	0.00% (0)	36.40% (4)	0.090
Supine HR > 75 bpm	0.00% (0)	27.30% (3)	0.214
Supine SBP, mmHg	114 (107; 127)	132 (124; 140)	0.198
Supine DBP, mmHg	74 (68; 83)	89 (82; 100)	0.065
Low supine BP [< 110/70 mmHg]	18.20% (2)	9.10% (1)	1.000
3 min tilt HR, bpm	68 (65; 73)	73 (69; 76)	0.129
3 min tilt SBP, mmHg	99 (81; 113)	105 (96; 125)	0.235
3 min tilt DBP, mmHg	67 (64; 78)	79 (67; 88)	0.184
Δ 3 min tilt HR, bpm	7 (5; 12)	5 (2; 7)	0.222
Δ 3 min tilt SBP, mmHg	− 26 (− 27; − 15)	− 21 (− 29; − 15)	0.851
Δ 3 min tilt DBP, mmHg	− 10 (− 16; − 2)	− 12 (− 16; − 9)	1.000
Δ 3 min BP fall [> 30/15 mmHg]	27.30% (3)	27.30% (3)	1.000
Δ 3 min BP fall [> 40/20 mmHg]	18.20% (2)	18.20% (2)	1.000
Standing test			
Supine HR, bpm	59 (54; 65)	67 (64; 73)	0.033
Supine HR > 70 bpm	9.10% (1)	45.50% (5)	0.149
Supine HR > 75 bpm	9.10% (1)	18.20% (2)	1.000
Supine SBP, mmHg	128 (113; 139)	133 (122; 141)	0.961
Supine DBP, mmHg	81 (75; 92)	90 (74; 100)	0.504
Low supine BP [< 110/70 mmHg]	18.20% (2)	18.20% (2)	1.000
3 min standing HR, bpm	69 (67; 76)	77 (73; 80)	0.069
3 min standing SBP, mmHg	108 (99; 119)	97 (88; 115)	0.194
3 min standing DBP, mmHg	73 (69; 85)	70 (59; 80)	0.124
Δ 3 min standing HR, bpm	9 (4; 21)	9 (4; 14)	1.000
Δ 3 min standing SBP, mmHg	− 20 (− 40; − 1)	− 27 (− 43; − 14)	0.271
Δ 3 min standing DBP, mmHg	− 9 (− 19; 5)	− 19 (− 26; − 7)	0.067
Δ 3 min BP fall [> 30/15 mmHg]	36.40% (4)	63.60% (7)	0.395
Δ 3 min BP fall [> 40/20 mmHg]	27.30% (3)	45.50% (5)	0.659
Deep breathing			
*E*/*I* ratio	4 (3; 6)	3 (2; 4)	0.125
Pathological age-adjusted* E*/*I* ratio	90.90% (10)	90.90% (10)	1.000
Valsalva maneuver			
Valsalva ratio	1.16 (1.09; 1.23)	1.10 (1.07; 1.13)	0.395
Pathologic age-adjusted Valsalva ratio	36.40% (4)	54.50% (6)	0.670
Δ Valsalva P II_L—P II_E			
SBP, mmHg	− 1 (− 7; 0)	− 8 (− 17; − 4)	0.086
DBP, mmHg	− 5 (− 6; 0)	− 5 (− 12; − 1)	1.000
Mean BP, mmHg	− 3.63 (− 6.6; 0.33)	− 5.28 (− 12.87; − 1.98)	0.395
Missing P II_L—P II_E BP overshoot*	72.70% (8)	90.90% (10)	0.586
Δ Valsalva P IV—P I			
SBP, mmHg	− 8 (− 14; 0)	− 12 (− 24; − 9)	0.186
DBP, mmHg	− 14 (− 20; − 5)	− 13 (− 22; − 10)	0.609
Mean BP, mmHg	− 11.22 (− 18.48; − 5.94)	− 11.55 (− 19.80; − 9.90)	0.391
Missing P IV—P I BP overshoot*	81.80% (9)	100.00% (11)	0.476

Categorical variables are characterized as absolute *n* and relative (%, in parentheses) values; continuous variables are characterized as the median value and the 25–75th percentiles (in parentheses)

*PD* Parkinson’s disease, *MSA-P* parkinsonian variant of multiple system atrophy, *nSH* neurogenic supine hypertension, *HR* heart rate, *SBP* systolic blood pressure, *DBP* diastolic blood pressure, *BP* blood pressure, *min* minute, *E/I ratio* expiratory/inspiratory ratio, *P II_L* late phase II, *P II_E* early phase II, *P IV* phase IV, *P I* phase I

*Calculated based on mean BP values

## Discussion

In our study, we found that neither an expert-based blinded CAFT evaluation nor a systematic comparison of cardiovascular autonomic indices was able to distinguish MSA-P from PD once nOH is present. To our knowledge, this blinded assessment was the first attempt of its kind to challenge multiple experts with identifying the correct diagnosis of MSA-P or PD solely using CAFT findings and continuous trend monitoring. The inter-rater reliability, which was calculated as Fleiss’* κ* [39], was 0.01, indicating slight inter-rater agreement. A manual second step analysis of six correctly diagnosed PD and three MSA-P cases incorrectly diagnosed as PD revealed neither a characteristic pattern nor a distinct autonomic measure that would have permitted discrimination between PD and MSA. Interestingly, in the three misdiagnosed MSA-P cases, a lack of increase in HR after 3 min of tilt was observed, which contrasts with previous reports [10, 26, 28] and might explain the raters’ decision to assign a diagnosis of PD. Furthermore, we analyzed why each rater’s evaluation scheme failed to achieve a sufficiently accurate classification. Although rater #1 used two of the four items with the highest diagnostic accuracy in their evaluation scheme (supine HR > 70 bpm; age < 65 years), combining these items did not increase diagnostic accuracy, while the other items had a diagnostic accuracy of < 60%. The same scenario applied to rater #2′s evaluation scheme, as combining the items with the highest diagnostic accuracy in all possible combinations failed to increase the diagnostic accuracy (< 64%). Rater #3′s main rationale was based on the hypothesis that phase IV of the Valsalva maneuver is more blunted in PD than in MSA-P, but this did not show a reliable discriminatory capacity (diagnostic accuracy: 52.38%). However, the respective evaluation may have been limited by an absence of respiratory track records and artefact overlays within the printout of the TFM. Additional analysis of subjective rater impressions regarding their diagnostic decisions could not be performed due to missing source data. Despite our negative findings, the study provides novel insights; for instance, we found that combining multiple parameters of CAFT into a probability score can increase the diagnostic yield (Table 3).

As expected, the systematic analysis of clinical demographic characteristics showed that, despite their similar disease durations, MSA-P patients had a more advanced Hoehn and Yahr stage than the PD patients (*p* = 0.008). [1, 2]. While it was not found to correspond to a significant difference, nSH was present in only one PD patient but in almost half of the MSA patients. The analysis of cardiovascular parameters did not show any significant difference between the MSA-P and PD cohorts. However, due to the small sample size, false negative results cannot be ruled out.

In their study “Natural history of pure autonomic failure (PAF): a United States prospective cohort”, Kaufmann et al. [26] reported that a resting HR of > 70 bpm and a better preserved chronotropic response to tilt were associated with a future risk of phenoconversion of PAF into MSA rather than PD or dementia with Lewy bodies, but other studies have failed to distinguish MSA from PD on the basis of orthostatic HR changes [10]. A preserved HR increase in MSA patients compared to patients with PD was also reported by Pilleri et al. [28]. We did not observe a difference in chronotropic response upon tilt in the present study. However, the HR increase after 3 min of tilt was nonstatistically significantly lower in patients with MSA-P than in PD, possibly leading to the misdiagnosis of one in four MSA-P patients as PD patients during the blinded assessment. Nevertheless, our findings are in line with those of Norcliffe-Kaufmann et al. [10], who noted that the baroreflex gain index based on hemodynamic changes during the Valsalva maneuver as well as the chronotropic HR response after 3 min of tilt failed to show differences between MSA-P and PD. Moreover, our data are in line with previous studies suggesting that laboratory CAFT cannot distinguish between MSA and PD [17–20, 27]. In a recently published study of a large cohort of PD and MSA-P patients and subanalysis focusing on OH-positive parkinsonian patients only, Fanciulli et al. [27] showed that CAFT does not discriminate between MSA-P and PD, and they concluded that it is not the presence of OH but its early development that discriminates between MSA-P and PD (in conjunction with other urological autonomic features) [27, 41]. Other studies have reported that CAFT does allow MSA and PD to be differentiated; however, those studies included patients without and with cardiovascular autonomic failure, with the latter usually being more frequent in the MSA cohort than in the PD cohort [16, 21, 24, 25]. Interestingly, in their prospective study regarding the differentiation of MSA and PD, Lipp et al. [16] found that autonomic function testing enabled MSA to be distinguished from PD in a study population in which approximately 20% of the PD patients were affected by OH, whereas a comparison between PD and MSA patients affected by OH showed that they were indistinguishable based on BP monitoring. A similar observation was made by Vichayanrat et al. [32]. Moreover, variable CAFT protocols or standard operating procedures (e.g. drug intake, room setting) likely affect comparisons between studies. Although it is generally assumed that autonomic failure develops earlier and is more severe and frequently observed in MSA than in PD [11, 16], high variability in clinical presentation makes misdiagnosis highly possible [42]. Thus, studies investigating cardiovascular autonomic function in α-synucleinopathies should stratify for the presence of cardiovascular autonomic failure, as homogeneous study populations are required for comparative analysis.

## Conclusion

CAFT represents the gold standard in assessing the presence and severity of cardiovascular autonomic failure, but the observation that neither the blinded assessment nor the systematic comparison of cardiovascular autonomic function indices permitted reliable discrimination suggests that CAFT might be of limited value in the differential diagnosis of MSA-P and PD once nOH is present. However, the retrospective design, the absence of respiratory track records, artefact overlays on the printout of the TFM, the lack of a neuropathologically confirmed diagnosis, and—above all—the small sample size represent limitations of the present study that warrant replication of the study with a larger, independent sample.
